# Mechanical synthesis of chemically bonded phosphorus–graphene hybrid as high-temperature lubricating oil additive†

**DOI:** 10.1039/c7ra11691h

**Published:** 2018-01-25

**Authors:** Xinhu Wu, Kuiliang Gong, Gaiqing Zhao, Wenjing Lou, Xiaobo Wang, Weimin Liu

**Affiliations:** State Key Laboratory of Solid Lubrication, Lanzhou Institute of Chemical Physics, Chinese Academy of Sciences Lanzhou 730000 PR China wjlou@licp.cas.cn gqzhao@licp.cas.cn; University of Chinese Academy of Sciences Beijing 100049 PR China; Qingdao Center of Resource & New Materials Qingdao 266000 PR China

## Abstract

Red phosphorus (P) was covalently attached to graphene nanosheets (Gr) using high-energy ball-milling under a nitrogen atmosphere. Benefiting from the formation of phosphate and P–O–C bonds on graphene surfaces, the resulting phosphorus–graphene (P–Gr) hybrids exhibited excellent dispersion stability in polyalkylene glycol (PAG) base oil compared with graphene. Moreover, tribological measurement indicated that addition of 1.0 wt% P–Gr into PAG resulted in significant reduction in friction coefficient (up to about 12%) and wear volume (up to about 98%) for steel/steel contact at 100 °C, which was likely due to the formation of a boundary lubrication film on the sliding surfaces during the friction and wear processes. XPS analysis demonstrated that the tribofilm is composed of FeO, Fe_3_O_4_, FeOOH, FePO_4_, and the compounds containing C–O–C and P–O bonds.

## Abstract 2

Red phosphorus (P) was covalently attached to graphene nanosheets (Gr) using high-energy ball-milling under a nitrogen atmosphere.

## Graphical abstract

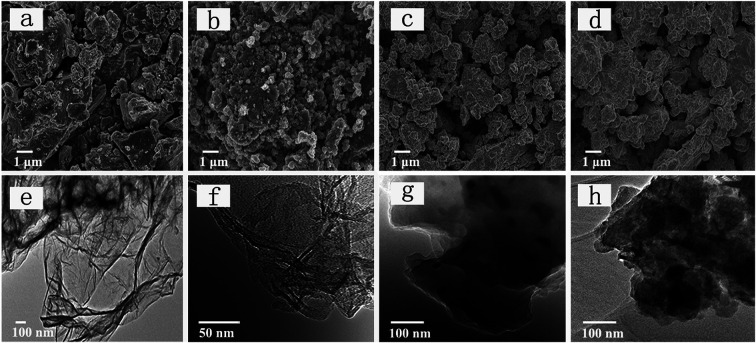

## 1. Introduction

Many different types of phosphorus-containing molecules have been investigated as additives for lubricating oils, with most attention given to their potential as friction reduction and antiwear (AW) additives. For instance, neutral triaryl phosphate (specifically tricresyl phosphate, TCP),^1^ metal-containing dithiophosphates (the predominant material of which is zinc dialkyldithiophosphates, ZDDPs),^2,3^ and the phosphorus-based ionic liquids (ILs).^4^ Because phosphorus-containing molecules can be film-forming at elevated temperatures,^5^ they are also used as high temperature lubricating oil additives. In recent years, our team have designed and synthesized a series of aryl phosphate, all of which possessed the advantages of high molecular weight, and high thermal and chemical stability, having been examined as high temperature oil additives.^5–7^ Additionally, a kind of tree-like polymeric phosphate esters (PPEs) covalently attached to graphene oxide (GO) nanosheets was also prepared and used in this application.^8^ However, tedious post-processing, large pollution and high cost are the main problems in a conventional preparation method of the phosphorus-based additives. Therefore, searching for new, cleaner synthetic methodologies has become increasingly important.

The major inspiration behind the rediscovery of mechanochemistry is green chemistry. Mechanochemistry refers to reactions, normally of solids, induced by the input of mechanical energy, such as by grinding in ball mills.^9–11^ Mechanical milling is a powerful tool among the various approaches for making solid materials. Recently, intensive research is carried out at formation of phosphorus/carbon hybrid by ball-milled as anode materials for sodium ion batteries (SIBs).^12–14^ In particular, Song *et al.*^12^ have reported that graphene stacks can be mechanically exfoliated to nanosheets and chemically bond with the surfaces of red phosphorus particles through a simple ball-milling approach, which also undoubtedly provided a cleaner and eco-friendly synthetic methodologies for phosphorus-based additives. Because this type of additives, regardless of composition, serve the same and specific function of bringing phosphorus into contact with the metal surface, where it can be adsorbed and, under certain conditions, react. The resulting surface film improves the lubrication properties of base oils. On the other hand, graphene, a layered two-dimensional (2D) crystal, always is presumed to be excellent lubricant additive.^15,16^ Its superthin, lightweight, large surface area and flexibility, make graphene suitable as an efficient substrate for improving dispersibility of phosphorus in lubricating oils.^17,18^ Moreover, the graphene nanosheets can chemically bond with phosphorus during the milling process, facilitating intimate contact between them and also preventing the aggregation of phosphorus.^12,19^

Considering the result mentioned above, in this paper, the low cost, environment-friendly starting materials of red phosphorus and graphene nanosheets together with the ball-milling approach make phosphorus–graphene (P–Gr) hybrid promising for practical application in lubrication field. The different weight ratios of red phosphorus (P) to graphene were prepared including 3 : 7, 1 : 1, and 7 : 3, and the products obtained were characterized by several microscopy and spectroscopic technique. The stability of P–Gr hybrid suspensions in polyalkylene glycol (PAG) base oil was evaluated, using PAG containing graphene (Gr) for comparison. Furthermore, the friction reducing and antiwear (AW) properties of these dispersions were investigated by a ball-on-disk apparatus at elevated temperature. The lubrication mechanism of P–Gr composite as additive in PAG was evidenced by scanning electron microscopy (SEM) and X-ray photoelectron spectroscopy (XPS).

## 2. Experiment section

### 2.1 Materials preparation

**Scheme 1:**
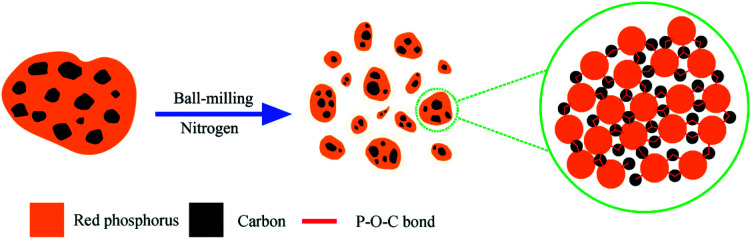
Schematic illustration of the fabrication of phosphorus–graphene nanosheets (P–Gr) hybrid and the formation of P–O–C bonds.

The synthesis of red phosphorus–graphene (P–Gr) hybrid was carried out according to the procedures described by Song *et al.*^12^ with some modifications. As shown in Scheme 1, the commercial red phosphorus (Alfa Aesar, 98.9%) and graphene flakes (Nanjing XFNANO Materials Tech Co. Ltd.), with mass ratios of 3 : 7, 1 : 1, and 7 : 3, were placed in a stainless steel jar and sealed in a glove box under nitrogen protection, followed by ball-milling for 16 hours at a speed of 300 rpm to obtain the resulting products: P–Gr (3 : 7), P–Gr (1 : 1) and P–Gr (7 : 3).

### 2.2 Structural characterizations

The morphology of P–Gr composite was investigated by field emission scanning electron microscopy (FESEM; Hitachi SU8020) and transmission electron microscopy (TEM; FEI TECNAI F30, 300 keV). The microstructure of the samples was characterized by powder X-ray diffraction (XRD; Bruker D8 DISCOVER) with Cu Kα radiation (*λ* = 1.54 Å). Raman spectra were recorded using a LabRAM-HR (Horiba) Raman microspectrometer with the excitation laser line of 514.5 nm. X-ray photoelectron spectroscopy (XPS) was conducted on a PHI-5702 X-ray photoelectron spectrometer using Al Kα radiation, and the binding energy of contaminated carbon (C 1s: 284.8 eV) was used as reference. Fourier transformation infrared (FT-IR) spectra were recorded on a Nicolet iS10 FT-IR spectrometer, using the KBr disk method. Thermogravimetric analysis (TGA) was carried out on a STA 449 F3 Jupiter simultaneous TG-DSC instrument from 25 to 800 °C at a heating rate of 10 °C min^−1^ under nitrogen flow.

### 2.3 Tribological characterization

Polyalkylene glycol (PAG) base oil used in the present investigation possesses the same typical physical characteristics as that used in our previous studies.^5^ PAG blends of various P–Gr hybrids with contents of 0.0, 0.1, 0.5, 1.0 and 1.5 wt% were made by magnetic stirring for 1 hour and thereafter sonicating for 30 min at room temperature. PAG with 1.0 wt% Gr was also prepared using the same method for comparison. The tribological measurements were evaluated on an Optimal-SRV-IV reciprocation friction tester with a ball-on-disk configuration. The upper ball (*Ø* 10 mm, AISI 52100 bearing steel) slides reciprocally at an amplitude of 1 mm against the stationary lower steel disk (AISI 52100 bearing steel, *Ø* 24.00 × 7.88 mm). Prior to the tribological test, 0.1–0.2 g lubricant (ASTM Method D-5707-98) was introduced to the ball–disk contact area. The friction coefficient was obtained by a computer program automatically. The wear volumes of the lower disks were measured using a MicroXAM-3D noncontact surface mapping profilometer. All the tests were repeated at least 3 times to confirm reproducibility of the results.

## 3. Result and discussion

### 3.1 Morphology and microstructure of P–Gr

**Fig. 1:**
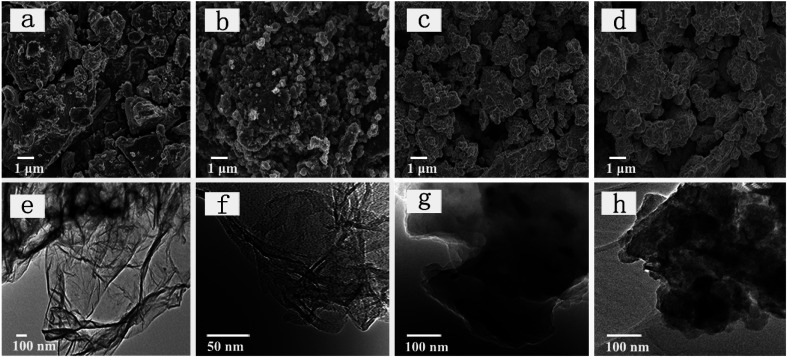
(a–d) FE-SEM images of red phosphorus (P), P–Gr (3 : 7), P–Gr (1 : 1) and P–Gr (7 : 3), and (e–h) TEM images of graphene, P–Gr (3 : 7), P–Gr (1 : 1) and P–Gr (7 : 3).

The SEM images of red P, P–Gr (3 : 7), P–Gr (1 : 1) and P–Gr (7 : 3) are presented in Fig. 1a–d. For the bulk commercial red P SEM image shows irregularly shaped particle with size up to a dozen microns (Fig. 1a). On the contrary, the P–Gr hybrid particles are found to be much smaller than the bulk phosphorus particles, ranging from submicron to a few microns in size, as shown in Fig. 1b–d. Moreover, the TEM images display that red P are uniformly distributed on graphene sheets (Fig. 1f–h), and the P–Gr hybrid layer thickness increased with increasing the content of red P compared with graphene sheets (Fig. 1e).

**Fig. 2:**
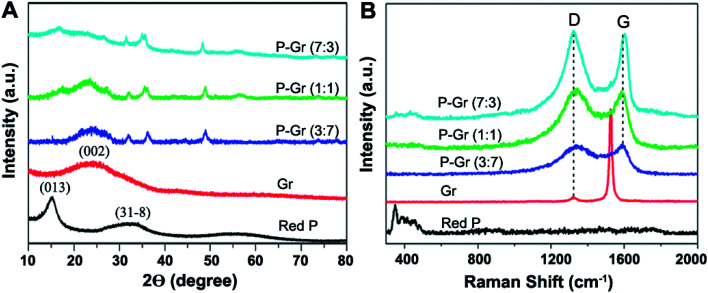
(A) XRD and (B) Raman spectra of the commercial red P, graphene and P–Gr hybrids.

Fig. 2 shows the X-ray diffraction (XRD) patterns and Raman spectra for red P, graphene (Gr), and P–Gr hybrid with different P/Gr mass ratio. The XRD patterns of the commercial red P shows a sharp diffraction peak at 15° (Fig. 2A), suggesting a medium-range ordered structure.^20^ A weak and broad peak at 2*θ* of 24.9° in the XRD pattern of the graphene stacks correspond to (002) planes of amorphous carbon. The broad diffraction peak of graphene can be due to the small size of the layers or a relatively short domain order of the stacked sheets, each of which broadens the XRD peak. When the hybrid of P–Gr with mass ratio of 3 : 7, 1 : 1 and 7 : 3 were prepared *via* ball-milling of red P with graphene for 16 h, there is a new peak at 2*θ* of around 48.4° in the XRD pattern of each hybrid corresponding to the compound with P–O bonds (JCPDS no. 85-0772), indicating partial oxidation of red P during the ball-milling process.

The structure of P–Gr hybrids is further investigated by the Raman spectra (Fig. 2B). Three bands from 300 to 500 cm^−1^ can be assigned to red P. The two peaks at 1326 cm^−1^ and 1530 cm^−1^ are the D-band and G-band of graphene, respectively. However, the native red P bonds disappear in the Raman spectra of P–Gr (3 : 7) and P–Gr (1 : 1) hybrids, and the intensity of these bonds is significantly decreased in that of P–Gr (7 : 3) hybrid, which is due to the breakup of some P–P bonds for generating the P–O–C bonds.^13^ Moreover, in comparison with the graphene stacks, the intensity ratio of the D band to the G band (*I*_d_/*I*_g_) in P–Gr (3 : 7), P–Gr (1 : 1) and P–Gr (7 : 3) hybrids increased from 0.057 to 0.98, 1.0 and 1.02, respectively, suggesting an increase in the number of sp^3^ carbons that were formed on the graphene during the ball-milling process.^21^ Also, the wave shift of the G band from 1530 cm^−1^ to about 1596 cm^−1^ indicates the decreased layers of graphene in P–Gr hybrids.

**Fig. 3:**
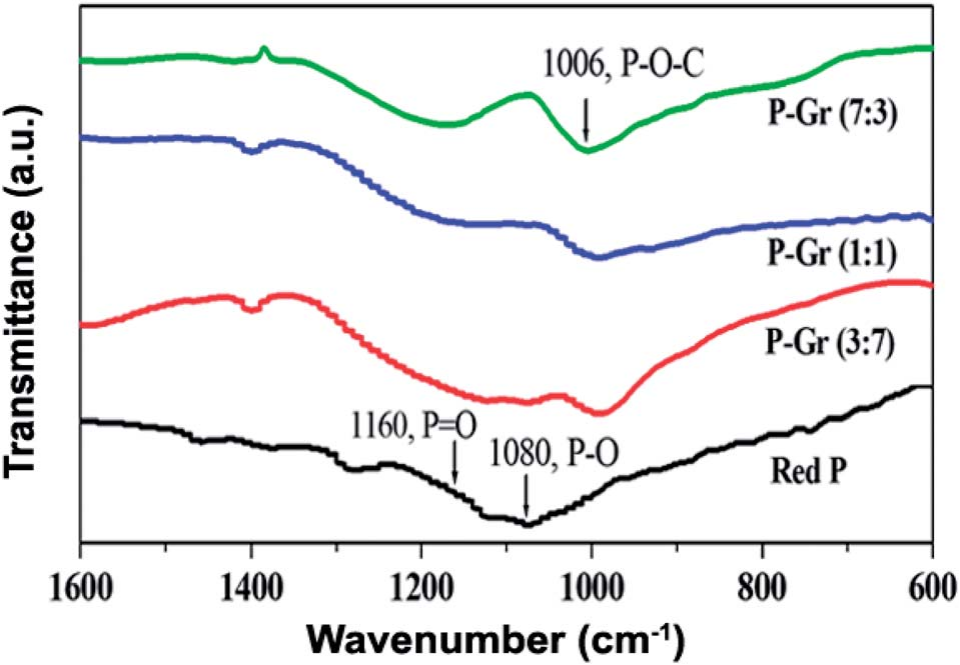
FT-IR spectra of red P, P–Gr (3 : 7), P–Gr (1 : 1) and P–Gr (7 : 3).

**Fig. 4:**
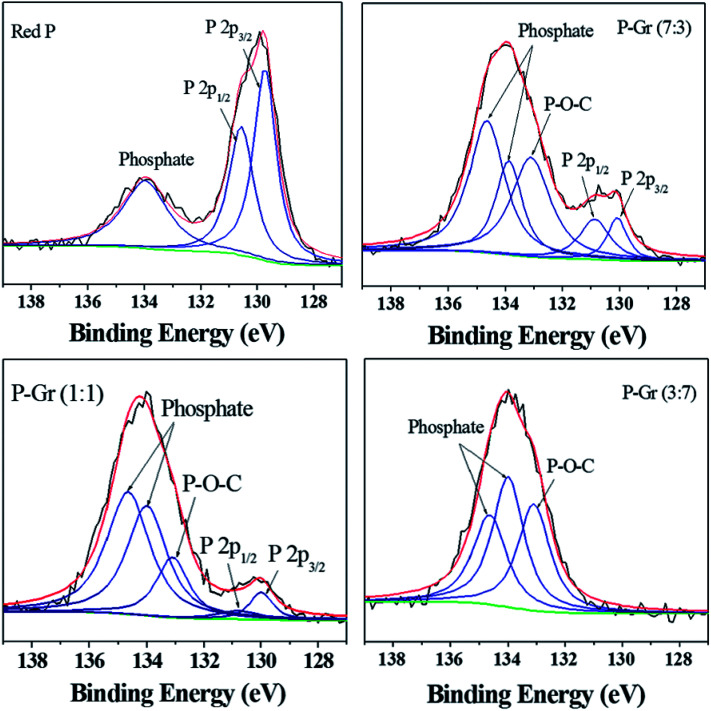
High-resolution XPS spectra of P 2p for red P, P–Gr (7 : 3), P–Gr (1 : 1) and P–Gr (3 : 7).

The formation of P–O–C bond in the hybrids of P–Gr with different P/Gr mass ratio was further investigated by FT-IR and XPS analysis. As shown in Fig. 3, the pristine red P shows two peaks centered at 1080 and 1160 cm^−1^, correlating to the P–O and P=O bonds.^12^ In contrast, a new signal located at about 1006 cm^−1^ is observed for all the hybrids, with the signal intensity of P–O and P=O decrease. This result can give an indication of the generation of P–O–C binds during ball-milling. Fig. 4 shows the P 2p XPS spectra of red P and the hybrids of P–Gr. The XPS spectra of red P has been fitted to three peaks located at 129.7, 130.5 and 134.0 eV. The peaks at 129.7 and 130.4 eV can be assigned to P 2p_3/2_ and P 2p_1/2_, respectively.^12^ The broad peak at 134.0 eV is assigned to phosphate.^22^ It should be noted that in the P 2p XPS spectra of P–Gr, an additional shoulder peak at ∼133.1 eV appeared. The position of this peak suggests the presence of P–O–C bonds.^12^ Moreover, it is seen that the signal intensity of phosphate increased with increasing the content of Gr while the P 2p component corresponding to P 2p_3/2_ and P 2p_1/2_ decrease. The results from XPS imply that red P have been successfully covalent attached to graphene through P–O–C bonds.

**Fig. 5:**
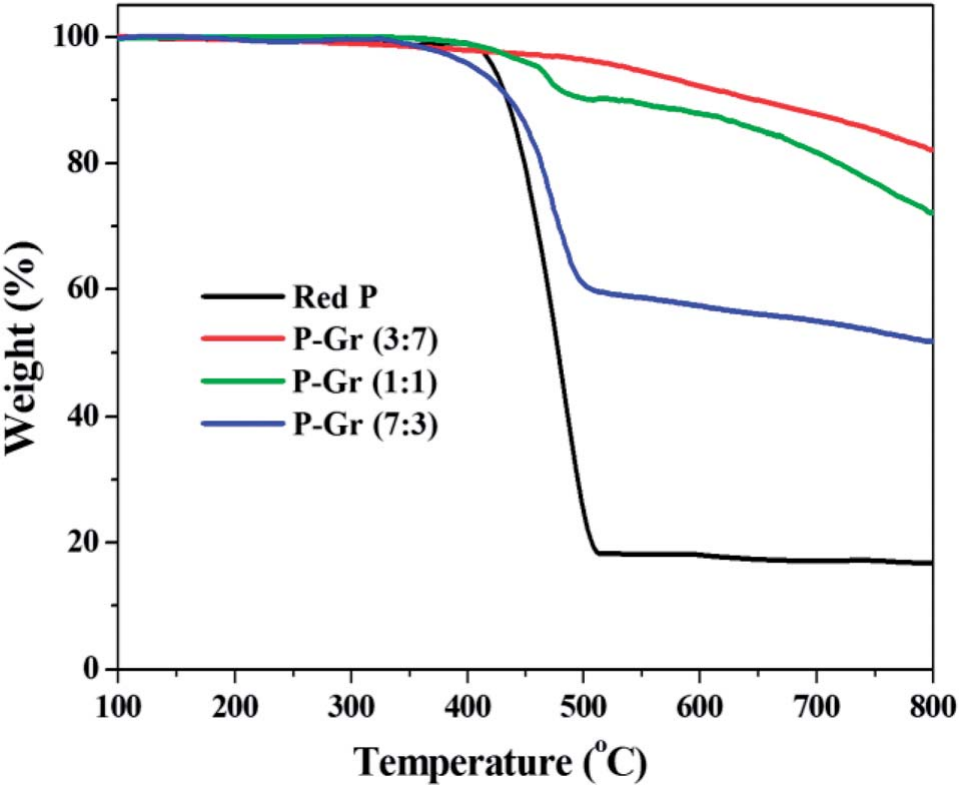
TGA thermograms of red P, P–Gr (3 : 7), P–Gr (1 : 1) and P–Gr (7 : 3) in nitrogen environment at a heating rate of 10 °C min.

Fig. 5 shows the TGA thermogram of red P and the hybrids of P–Gr with different P/Gr mass ratios. It is seen that the decomposition temperatures (*T*_d_) of P–Gr (3 : 7), P–Gr (1 : 1) and P–Gr (7 : 3) are 525, 432, and 403 °C, respectively, indicating high thermal stability of P–Gr hybrids. Moreover, all of the hybrids exhibit high thermal stability compared to the pristine red P, which is most likely due to the formation of P–O–C binds between red P and graphene *via* high-energy ball milling.

### 3.2 Friction and wear behavior of P–Gr hybrid added in PAG

**Fig. 6:**
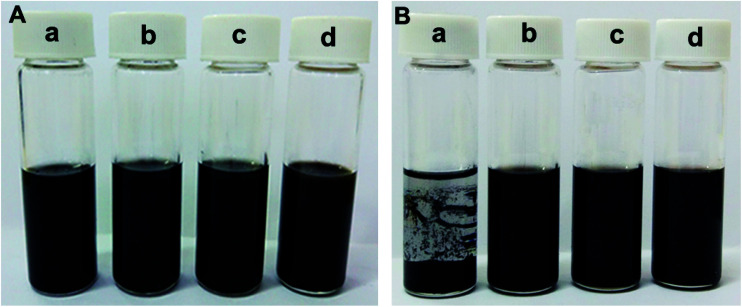
Photograph of PAG containing (a) 1.0 wt% Gr, (b) 1.0 wt% P–Gr (3 : 7), (c) 1.0 wt% P–Gr (1 : 1), and 1.0 wt% P–Gr (7 : 3). The hybrid lubricants were equilibrated for (A) one month and (B) two months after preparation, respectively.

**Fig. 7:**
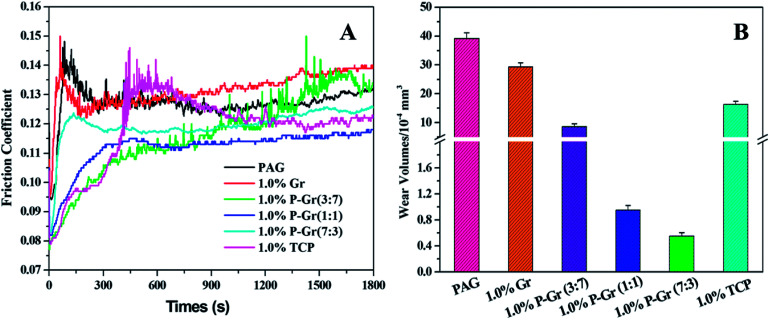
(A) Friction coefficient and (B) wear volumes of the disks lubricated by PAG and PAG containing 1.0 wt% Gr, 1.0 wt% P–Gr (3 : 7), 1.0 wt% P–Gr (1 : 1), 1.0wt% P–Gr (7 : 3) and 1.0 wt% TCP at 100 °C (SRV load, 100 N; duration, 30 min; stroke, 1 mm; frequency, 25 Hz).

All of the above additives show exceptional stability in PAG base oil, and the dispersion stability of PAG containing 1.0 wt% P–Gr (3 : 7), 1.0 wt% P–Gr (1 : 1), and 1.0 wt% P–Gr (7 : 3) was found to be stale and resist sedimentation for at least two month after physical dispersion treatments (shown in Fig. 6b–d), while the addition of 1.0 wt% Gr fails to disperse (Fig. 6a), which might due to the formation of phosphate and P–O–C bonds on graphene surfaces. The lubricating performances of PAG containing different content of P–Gr were evaluated using a ball-on-disk reciprocating configuration at 100 °C. The normal load was 100 N, the oscillation frequency was 25 Hz with 1 mm amplitude. The results indicate that the addition of 1.0 wt% P–Gr in PAG is the optimum content to provide good friction reduction and AW properties for steel/steel contacts at 100 °C (Fig. S1–S3†). For comparison, Fig. 7 displays the friction coefficient and wear volumes of pure PAG, and PAG plus 1.0 wt% Gr, 1.0 wt% P–Gr (3 : 7), 1.0 wt% P–Gr (1 : 1), 1.0 wt% P–Gr (7 : 3) and 1.0 wt% tricresyl phosphate (TCP) obtained under the same conditions. As shown in Fig. 7A, PAG exhibits a relatively high friction coefficient with an average value of 0.127, and addition of 1.0 wt% Gr leads to a slight increase in the friction coefficient. For PAG containing 1.0 wt% P–Gr (3 : 7), P–Gr (1 : 1), P–Gr (7 : 3) and TCP, the friction coefficients achieved are 0.116, 0.112, 0.120 and 0.122, which are 9%, 12%, 6% and 4% reduction relative to the pure PAG, respectively. Moreover, the addition of 1.0 wt% Gr, P–Gr (3 : 7), P–Gr (1 : 1), P–Gr (7 : 3) and TCP can reduce the wear by around 25%, 76%, 97%, 98% and 58% compared to the base oil, respectively (Fig. 7B). These results demonstrated that P–Gr hybrids have exceptional friction reduction and AW behaviors. Overall, there appears to be a general trend that with increasing the content of phosphorus in P–Gr hybrids, the AW property increases. In addition, the hybrid with higher content of phosphorus shows a relatively stable friction coefficient compared to that with lower content of phosphorus. The results can be explained by the fact that a thick, patchy boundary film that maintains low friction and wear was formed on the rubbing surfaces lubricated by P–Gr with high content of phosphorus in PAG, while the boundary film might be worn away and could not complement rapidly as the hybrid of P–Gr with low content of phosphorus was used as additive in the base oil.

**Fig. 8:**
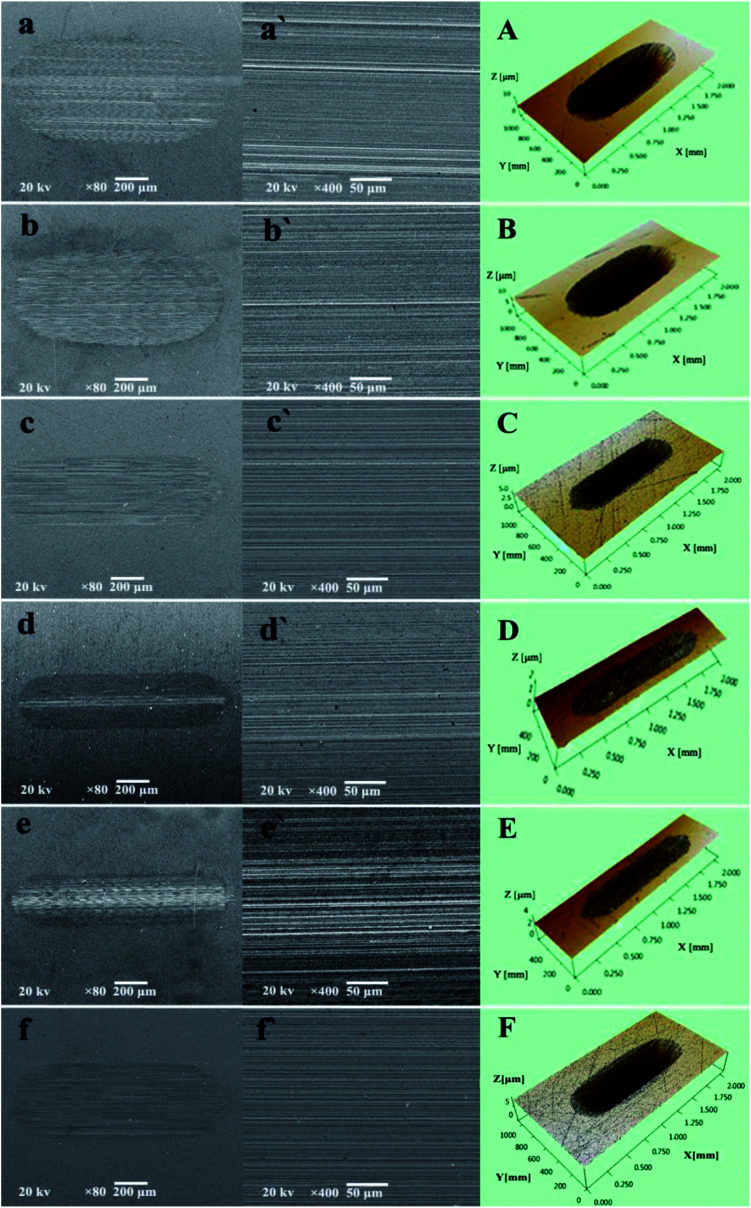
(aa′–ff′) Scanning electron microscope (SEM) and (A–F) three-dimensional (3D) optical microscopic images of the worn surfaces lubricated by pure PAG, and PAG with 1.0 wt% Gr, 1.0 wt% P–Gr (3 : 7), 1.0 wt% P–Gr (1 : 1), 1.0 wt% P–Gr (7 : 3) and 1.0 wt% TCP at 100 °C (SRV load, 100 N; duration, 30 min; stroke, 1 mm; frequency, 25 Hz).

The excellent AW property of P–Gr hybrids were further checked with scanning electron microscope (SEM) and three-dimensional (3D) surface mapping profilometer. As can be seen from Fig. 8aa′ and bb′ that the wear scars lubricated by PAG and 1.0 wt% Gr are very wide and deep, with a number of deep and narrow grooves, indicating severe wear. However, the addition of 1.0 wt% P–Gr (3 : 7) and 1.0 wt% TCP can make the wear scar becomes relatively narrow and shallow (Fig. 8cc′ and ff′), showing that both P–Gr (3 : 7) and TCP have some certain AW property. In contrast, worn surfaces under the lubrication of PAG containing 1.0 P–Gr (1 : 1) (Fig. 8d) and 1.0 wt% P–Gr (7 : 3) (Fig. 8e) show very little wear with worn surfaces dramatically becoming narrow and shallow. Meanwhile, Fig. 8A–F show the 3D morphology of wear scars, which clearly displays the wear scenario under lubrication of PAG and PAG plus different additives. These results are in accordance with the wear volumes shown above (Fig. 7B), and again demonstrates that P–Gr additive can greatly improve the AW property of pure PAG for steel/steel contact at elevated temperature.

**Fig. 9:**
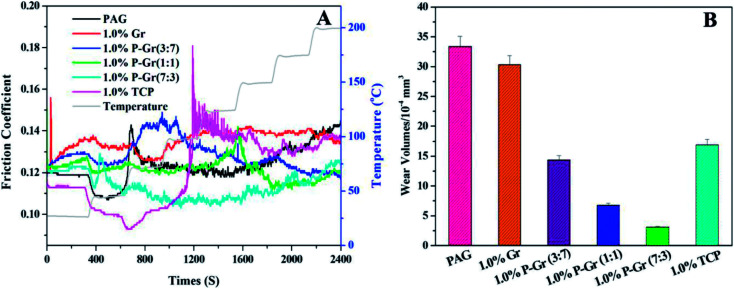
(A) Variations of the friction coefficient with time during a temperature ramp test from 25 to 200 °C and (B) the corresponding wear volumes for PAG and PAG plus 1.0 wt% P–Gr (3 : 7), 1.0 wt% P–Gr (1 : 1), 1.0 wt% P–Gr (7 : 3) and 1.0 wt% TCP at a constant load of 100 N, and a frequency of 25 Hz.

To illustrate the effect of temperature on tribological properties, Fig. 9 shows a temperature ramp test from 25 up to 200 °C stepped by 25 °C at 100 N for PAG and PAG containing 1.0 wt% Gr, P–Gr (3 : 7), P–Gr (1 : 1), P–Gr (7 : 3) and TCP. The test duration for each load was 5 min. It can be seen that all the additives show a marked increases in the friction coefficient compared to the base oil at a temperature below 50 °C, partially owing to the relative high viscosity of base oil with additives (Fig. 9A). When the temperature is higher than 50 °C, the addition of these additives, except for 1.0 wt% Gr and P–Gr (3 : 7), exhibit better friction reducing behavior than the neat PAG. Furthermore, the wear volumes of PAG containing different additives increase in the following sequence: 1.0 wt% P–Gr (7 : 3) < 1.0 wt% P–Gr (1 : 1) < 1.0 wt% P–Gr (3 : 7) 1.0 wt% TCP < 1.0 wt% Gr < PAG (Fig. 9B). The good performance of phosphorus-based additives at elevated temperature has been explained in the previous section, and the results further confirm that P–Gr hybrids with high content of phosphorus will lead to significant reduction in friction coefficient and wear volume at elevated temperature.

### 3.3 Surface analysis

**Fig. 10:**
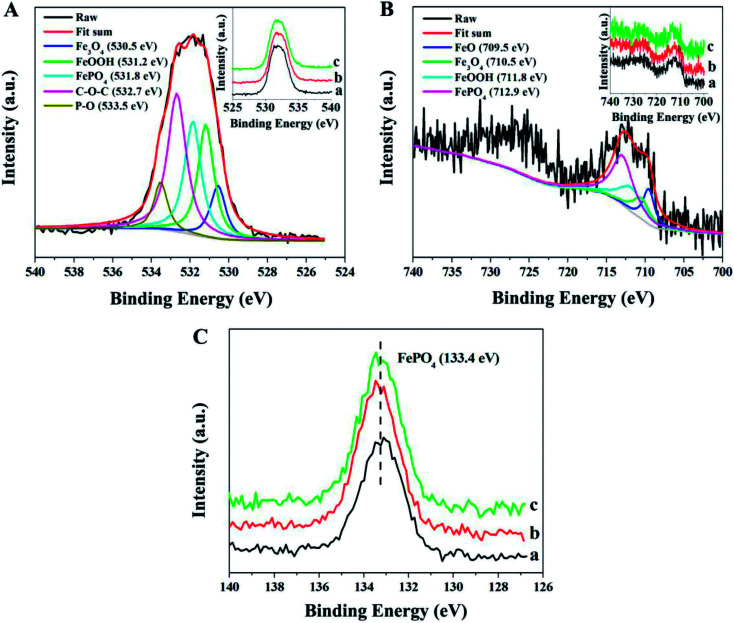
XPS spectra of O 1s, Fe 2p, and P 2p of the worn surfaces lubricated by PAG containing (a) 1.0 wt% P–Gr (3 : 7), (b) 1.0 wt% P–Gr (1 : 1) and (c) 1.0 wt% P–Gr (7 : 3) at 100 °C (SRV load, 100 N; duration, 30 min; stroke, 1 mm; frequency, 25 Hz).

The tribochemical reaction products forming at the contact interface were detected by XPS spectra in order to explore the friction reduction and AW properties of P–Gr in PAG. Fig. 10 shows the XPS spectra of worn steel disk surfaces lubricated by PAG containing different P–Gr hybrids at 100 °C. It is found that the binding energies of O, Fe and P of the worn surfaces lubricated by 1.0 wt% P–Gr (3 : 7), P–Gr (1 : 1) and P–Gr (7 : 3) at 100 °C are similar to each other, indicating that the hybrids with different P/Gr mass ratios on the worn surface had similar tribochemical reactions. This is due to the fact that phosphorus-based additives, regardless of composition, serve the same and specific function of bringing phosphorus into contact surface to boundary lubrication film that improves the lubrication properties of base oils. The XPS spectra of O 1s has five peaks at 530.5, 531.2, 531.8, 532.7 and 533.5 eV corresponding to Fe_3_O_4_, FeOOH, FePO_4_, C–O–C and P–O bonds,^5,23,24^ respectively (Fig. 10A). Fig. 10B reveals that the XPS spectra of Fe 2p appear at 709.5, 710.5, 711.8 and 712.9 eV, which might be attribute to FeO, Fe_3_O_4_, FeOOH and FePO_4_.^5–7,23^Fig. 10C shows the peaks of P 2p at about 133.4 eV, which can be assigned to FePO_4_.^24–26^ XPS analysis reveals that under harsh tribological testing conditions, complex mechano-chemical reactions, which involved the active element P of the P–Gr hybrid and the fresh-metal surface, occurred, generating a tribofilm composed of FeO, Fe_3_O_4_, FeOOH, FePO_4_, and compound containing C–O–C and P–O bonds on the wear scar. The film provided a protective boundary for the underneath material, thereby reducing friction and wear.

## 4. Conclusions

Phosphorus–graphene hybrids (P–Gr) with different P/Gr mass ratios were fabricated by mechanochemical reaction in a ball-milling process. This process produces P–O–C bonds, which are beneficial to the thermal stability of P–Gr. All of the hybrids P–Gr could be well dispersed in PAG base oil and the resulted dispersions are stable for more than two months. The tribological tests indicated that good friction reduction and significant improvement of wear behavior were observed with the addition of 1.0 wt% P–Gr in PAG for steel/steel contact at elevated temperature. A tribochemical reaction between the active element P of the P–Gr hybrid and the iron/iron oxide occurred during the friction and wear process, leading to the formation of a tribofilm composed of FeO, Fe_3_O_4_, FeOOH, FePO_4_, and compound containing C–O–C and P–O bonds on the wear scar. The tribofilm attributed the excellent friction reducing and AW performances of P–Gr hybrid in PAG base oil.

## Conflicts of interest

The authors declare no competing financial interest.

## Supplementary Material

RA-008-C7RA11691H-s001
