# Supplementary material for: Mechanical synthesis of chemically bonded phosphorus–graphene hybrid as high-temperature lubricating oil additive
Source: RSC Adv. 2018 Jan 25;8(9):4595–603. doi: 10.1039/c7ra11691h (PMC9077827; doi:10.1039/c7ra11691h)
Supplement: RA-008-C7RA11691H-s001 [file RA-008-C7RA11691H-s001.pdf]

Electronic Supplementary Material (ESI) for RSC Advances.
This journal is © The Royal Society of Chemistry 2018

Electronic Supplementary Information

Mechanical synthesis of chemically bonded phosphorus-graphene hybrid as high-temperature lubricating oil additive

X. H. Wu, K. L. Gong, G. Q. Zhao, W. J. Lou, X. B. Wang, and W. M. Liu

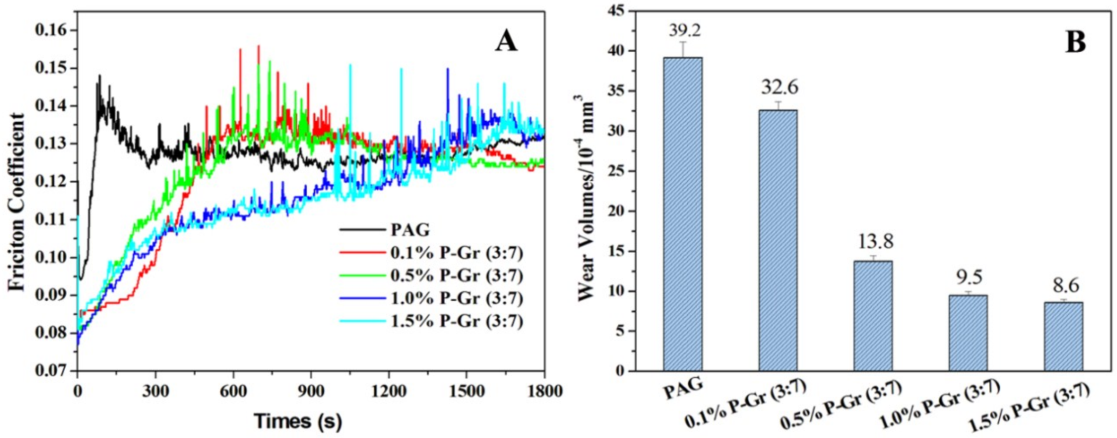

Fig. S1. (A) Friction coefficient and (B) Wear volumes of the discs lubricated by PAG and PAG containing different contents of P-Gr (3:7) at 100 ^{o}C (SRV load, 100 N; duration, 30 min; stroke, 1 mm; frequency, 25 Hz).

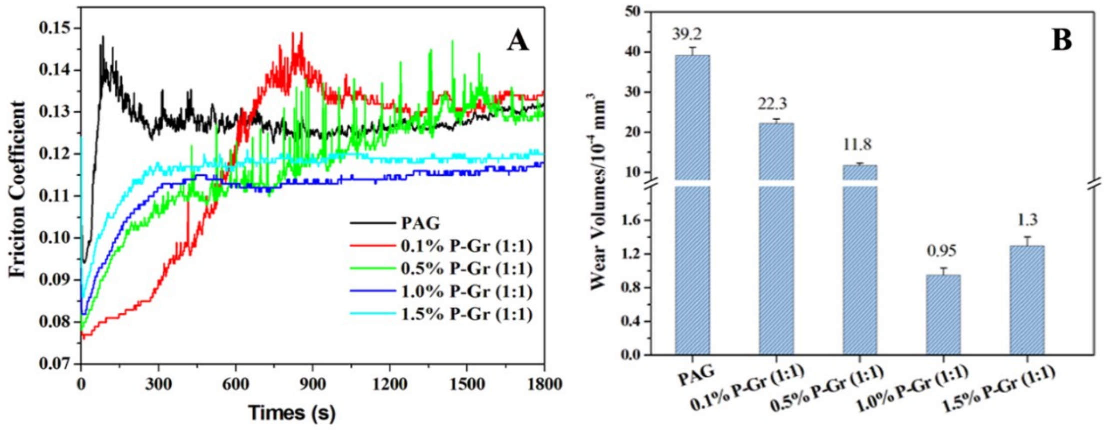

Fig. S2. (A) Friction coefficient and (B) Wear volumes of the discs lubricated by PAG and PAG containing different contents of P-Gr (1:1) at 100 ^{o}C (SRV load, 100 N; duration, 30 min; stroke, 1 mm; frequency, 25 Hz).

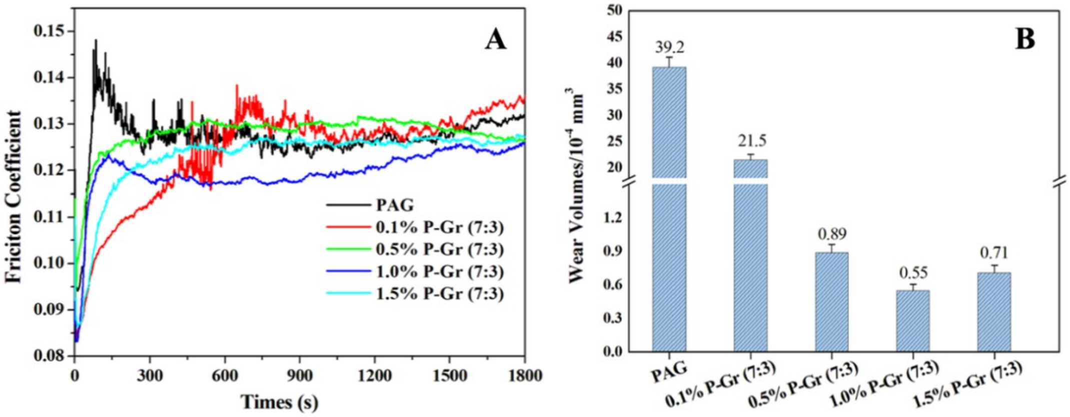

Fig. S3. (A) Friction coefficient and (B) Wear volumes of the discs lubricated by PAG and PAG containing different contents of P-Gr (7:3) at 100 ^{o}C (SRV load, 100 N; duration, 30 min; stroke, 1 mm; frequency, 25 Hz).
